# Microbiota of preterm infant develops over time along with the first teeth eruption

**DOI:** 10.3389/fmicb.2022.1049021

**Published:** 2022-12-22

**Authors:** Yu Zhang, Yi-Pei Wu, Vivien Feng, Gui-Zhi Cao, Xi-Ping Feng, Xi Chen

**Affiliations:** Shanghai Key Laboratory of Stomatology, Department of Preventive Dentistry, Shanghai Ninth People's Hospital, College of Stomatology, National Clinical Research Center for Oral Diseases, Shanghai Jiao Tong University School of Medicine, Shanghai Research Institute of Stomatology, Shanghai, China

**Keywords:** infants, preterm birth, flora microorganisms, saliva, teeth eruption

## Abstract

**Objective:**

The temporal growth of the infant microbiome in the early years of life influences short- and long-term infant health. The aim of this longitudinal study was to investigate bacterial dynamics in the microbiome of preterm infants during tooth eruption.

**Methods:**

Saliva samples from normally delivered (*n* = 24) and preterm infants (*n* = 31) were collected 30 days after birth and after the eruption of two primary mandibular incisors. Based on Illumina MiSeq Sequencing of the 16S rRNA gene, the dynamic microbial changes of newborns at two-time points were investigated. Meanwhile, the Human Oral Microbiome Database was adopted for assigning taxonomy.

**Results:**

Using alpha and beta diversity analyses, different shift patterns of microbiome structures in preterm and healthy participants and bacterial diversity over time were observed. The relative abundance and shifts trend, along with the two lower primary central incisors eruption, of core oral flora varies in full-term and preterm groups, including *Gemella* spp., *Rothia mucilaginosa, Veillonella atypica*, etc. Several microorganisms colonize later in the oral microbiome development of premature babies, such as *Gemella* spp. In addition to teeth eruption, the growth of the saliva microbiome in preterm infants could be influenced by breastfeeding durations and birth weight.

**Conclusion:**

This study provided insights into how the oral microbiota changes during tooth eruption in preterm infants and how the colonization of the oral cavity with bacteria in preterm infants differs significantly from that in full-term infants.

## Introduction

The human oral cavity refers to a complex microbial community consisting of bacteria, fungi, viruses, archaea, and protozoa, and the microorganisms constantly interact with each other (Lamont et al., 2018). Eubiosis of the oral microbiome is characterized by the “healthy oral microbiota” with specific microbial patterns, and the oral microbiome lives in homeostasis with the host. Moreover, changes in the balance of the oral microbiome cause dysbiosis, which leads to a pathogenic state and the occurrence and progression of oral diseases (Jiao et al., 2014). Microbial structure in the oral cavity is dependent on niche and age (Xu et al., 2015, 2022). Oral microbial maturation is triggered by age-associated biological alterations (Crielaard et al., 2011), whereas oral microbial communities formed during early life can affect the long-term systemic and oral health of a child (Dzidic et al., 2018).

The mother plays a crucial role in shaping the oral environment in early life (Sampaio-Maia and Monteiro-Silva, 2014; Teng et al., 2015; Dzidic et al., 2018), which subsequently develops into a complicated and mature microbial ecosystem due to parental vertical transmission and the external environment (Hesselmar et al., 2013; Sampaio-Maia and Monteiro-Silva, 2014). Almost all previous studies have classified diverse oral microbial developmental stages on the basis of age in months while simultaneously ignoring the effect brought by tooth eruption state. Typically, the eruption of the primary incisor starts at approximately 6 months after birth. It is of great importance to evaluate the influence of tooth eruption on the course of microbial colonization.

Preterm birth has been a major factor resulting in neonatal death worldwide and leading to higher risks for poor neurobehavioral development, metabolic disorders, and respiratory distress in neonates and mothers. Low birth weight in preterm infants has been associated with pathogenic bacteria in the oral cavity (Han and Wang, 2013; Cobb et al., 2017; Ye et al., 2022). Thus, higher detection frequencies of periodontal-associated microbial species, including *Porphyromonas gingivalis*, *Treponema denticola*, *Fusobacterium nucleatum*, *Prevotella intermedia*, and *Tannerella forsythia*, in saliva or dental plaque samples have recently been shown to be strongly associated with preterm low birth weight (Swati et al., 2012; Ye et al., 2022). Moreover, *P. gingivalis* infection-induced preterm birth as well as low birth weight in pregnant mice (Ao et al., 2015). In addition, it was considered that dysbiosis in the placental microbiome showed a relationship to preterm birth. To date, the impact of preterm birth on the development of the early oral microbiota has not been thoroughly investigated in longitudinal studies.

There are more and more studies on the interactions between the oral microbiota of mother and child and on the progressive development of the intestinal microbiota of infants (Backhed et al., 2015; Gomez and Nelson, 2017; Hill et al., 2017; Mason et al., 2018; Yang et al., 2020; Kaan et al., 2021). However, the changes observed in the oral microbiome and the progressive development of the infants’ microbiome during teeth eruption are not well understood. To date, there is no consensus on the characteristics of the oral microbiome colonization of preterm infants after birth.

On the basis of this, we aimed to characterize the dynamic shift in microbial structure and external factors associated with the microbiome of preterm infants during teeth eruption. In this longitudinal study, we collected saliva samples from normally delivered and preterm infants during the eruption of deciduous teeth and examined the dynamic microbial changes of the newborns at two time points using Illumina MiSeq sequencing of the 16S rRNA gene.

## Materials and methods

### Study design and samples

The work was supported by the Ethics Committee at the Ninth People’s Hospital, School of Medicine, Shanghai Jiao Tong University, Shanghai, China (Ref#: 2015135). Before recruitment and routine examination, informed consent was acquired from all parents of the enrolled infants. Complete clinical examinations of all infants included were performed, and their caregivers were asked to complete the questionnaire at recruitment and follow-up. We also followed specific regulations and institutional guidelines.

This longitudinal study was conducted at Children’s Hospital of Shanghai and Huinan Community Health Service Center, Department of Child Healthcare, Shanghai, China, from June 2018 to November 2020. The inclusion criteria for the healthy newborn group (CT group) included the following: (1) birth at full term (37–42 gestational weeks); (2) standard birth weight (2.5–4.5 kg); (3) age between 30 and 40 days at the time of enrollment; (4) no antibiotic use within 30 days; and (5) no oral or systemic disease during the entire period. The inclusion criteria for the preterm group (PT group) included: (1) gestational age between 28 and 35 weeks at birth; (2) low birth weight (less than 2.5 kg); (3) the age range of 30–40 days at the enrollment time; and (4) no present malformations or syndromes. All participating infants were followed up at 3, 6, 12, and 18 months of age until the eruption of deciduous teeth. All preterm infants included in the present work received the standard care offered by the hospital medical staff according to stability and degree of preterm birth.

Non-stimulated saliva specimens were collected during baseline and follow-up in this study. All infants were not allowed to eat or drink for 2 h before sampling. Under the assistance of one assistant and their parents, the same pediatric dentist was responsible for collecting oral samples. Saliva samples were first collected using two sterile cotton swabs that were carefully dipped into the saliva pool at the base of the mouth. Later, the tongue and buccal mucosa were gently wiped so that the saliva soaked the swab for approximately 60 s and transferred to an empty sterile Eppendorf tube.

**Figure 1:**
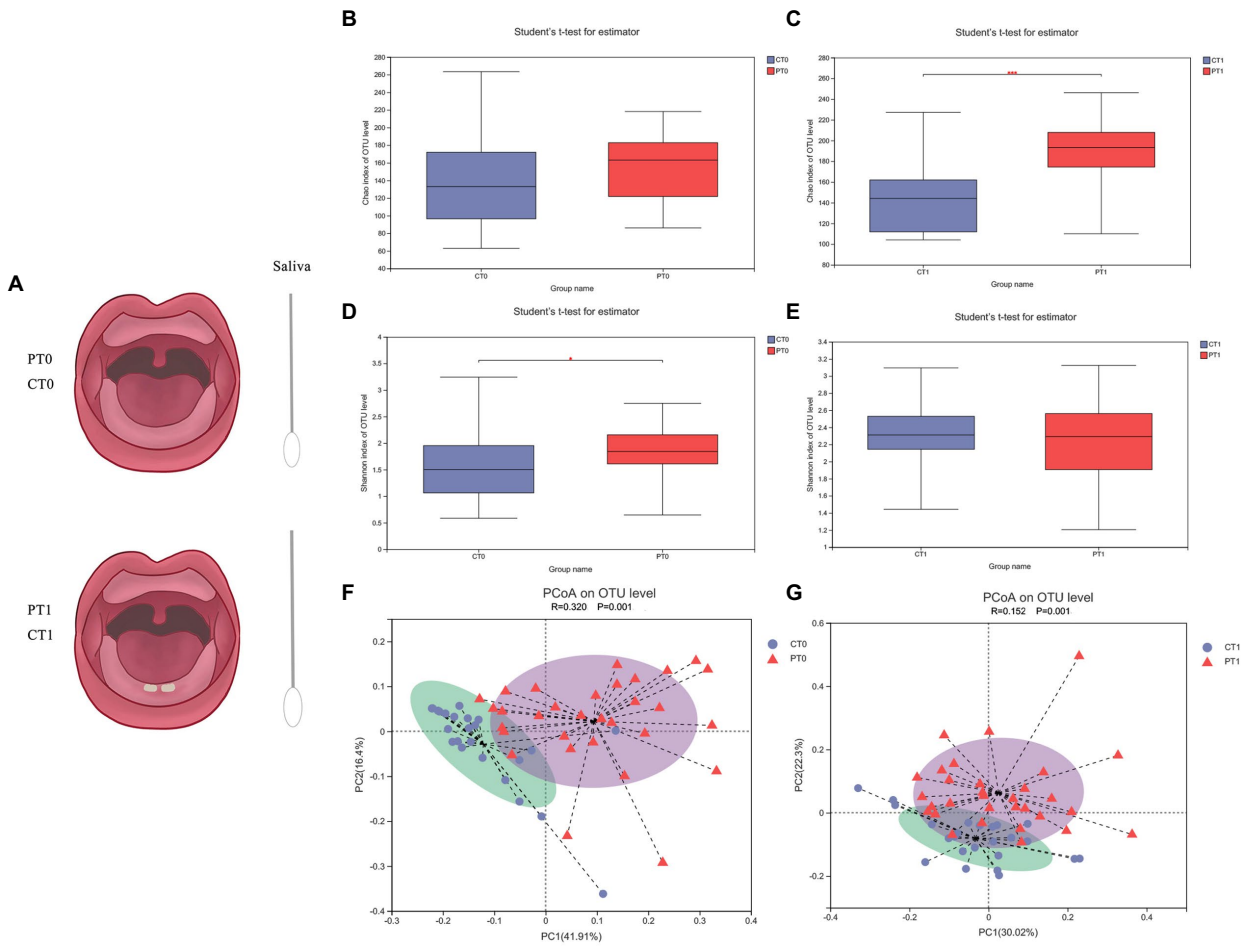
Flow chat and species diversity of microbiota in saliva samples of infants differ in preterm infants. **(A)** Flow chat of this study. **(B–E)** Bacterial richness and diversity (shown by Chao and Shannon indices), acquired at varying time points from the age of 1 month to teeth eruption, were identified using 16S rRNA Illumina sequencing together with OTU clustering at 97% concentration of sequence identity. *p < 0.05, ***p < 0.001. (**F,G)** PCoA of the weighted normalized UniFrac distance observed at OTU level for the bacterial community structure of the saliva microbiome between the healthy and preterm groups at two stages.

Among the 168 newborns meeting the inclusion criteria, salivary samples of 135 children were collected. During follow-up, 20 infants were excluded because they had a new-onset systemic disease or had taken antibiotics in the previous 30 days. A total of 15 children were not willing to be involved because of individual reasons, and 45 were absent during follow-up after the deciduous teeth eruption. Finally, a pool of saliva samples was obtained from these remaining infants in two specific dentition states (Figure 1A): first, saliva samples without tooth eruption were collected from 24 healthy newborns (CT0) and 31 preterm infants (PT0); and second, saliva samples with the eruption of the two mandibular central incisors were collected from corresponding 24 healthy newborns (CT1) and 31 preterm infants (PT1).

### DNA extraction and MiSeq sequencing

We collected the specimens in the morning (9–11 a.m.). In addition, all samples were immediately positioned in the bubble chamber, including dry ice for 4 h, which were subsequently kept at a − 80°C freezer. Using the Soil DNA Kit protocol (EZNA; Omega Bio-Tek, Norcross, GA, United States), microbial DNA was extracted from saliva samples. We also used the NanoDrop 2000 UV–Vis spectrophotometer (Thermo Fisher Scientific, Wilmington, MA, United States) to evaluate the purity and final content of the DNA. Subsequently, the quality of DNA was assessed with 1% concentration of agarose gel electrophoresis. Moreover, V3-V4 primers 338F (5′-ACTCCTACGGGAGGCAGCAG-3′), 806R (5′-GGACTACHVGGGTWTCTAAT-3′), and a thermocycler polymerase chain reaction (PCR) system (GeneAmp 9,700, ABI, United States) were adopted for amplifying bacterial 16S-rRNA gene (V3-V4 hypervariable segments). We also performed all experiments in triplicate with 20 μL aliquots containing 4 μL 5× FastPfu buffer, 2 μL 2.5 mM deoxynucleoside triphosphate, 0.8 μL of each primer (5 μM), 0.4 μL FastPfu polymerase, and 10 ng DNA template. In addition, the whole PCR process included the following steps: denaturation (3 min; 95°C, 27 × 30 s cycles at 95°C), annealing (30 s; 55°C), elongation (45 s; 72°C), and final extension (10 min; 72°C). This work employed 2% agarose gels for extracting PCR products as well as AxyPrep DNA Gel Extraction Kit (Axygen Biosciences, Union City, CA, United States), and QuantiFluor™-ST (Promega, Fitchburg, WI, United States) to perform further purification and calculation, respectively. Subsequently, we used Illumina MiSeq (Illumina, San Diego, CA, United States) for pooling the amplicons in equimolar paired-end sequencing (2 × 300) according to the instructions from Majorbio Bio-Pharm Technology Co Ltd. (Shanghai, China). The data presented in the study are deposited in the SRA repository, accession number PRJNA876796.

### Bioinformatics analysis

Each 16S-rRNA gene sequence was categorized with the RDP Classifier algorithm^1^ and explored against the Human Oral Microbiome Database (HOMD) database (v15.2). In addition, the representative sequences were categorized based on different classification levels (from phylum to species). Trimmomatic and FLASH software were used to interpret the sequencing data (Caporaso et al., 2010; Magoc and Salzberg, 2011), while UPARSE v7.1 software^2^ (Schloss et al., 2009) was used to cluster the operational taxonomic units (OTUs; similarity cutoff rate: 97%). Moreover, we used UCHIME (version 4.1) for detecting and obtaining the chimeric sequences.

On the basis of the alpha (α) index, the bacterial richness diversity of the saliva microbiota was evaluated. In this study, R-forge was used to assess beta diversity. Following the normalized weighted UniFrac distance matrices, a principal coordinate analysis (PCoA) was performed based on QIIME (Caporaso et al., 2010). Apart from that, this study analyzed similarities (ANOSIM) for evaluating the saliva microbiota composition of the participants. Moreover, using linear discriminant analysis (LDA) effect size (LEfSe), the relative abundance (mean differences) in species was assessed (Segata et al., 2011). Meanwhile, a fixed alpha value (0.05) was employed to perform the Kruskal-Wallis test. Apart from this, we set a threshold of 3.0 for the study of log LDA scores. The Wilcoxon rank sum test was used to compare the relative abundance of salivary microbiota between two participating groups with a false discovery rate for numerous adjustments. In addition, we measured the variance inflation factor (VIF) to evaluate the collinearity between the different environmental factors. At the same time, environmental elements having VIF >10 were eliminated from the correlation analysis below. The correlation of microbial community structure with influential elements was assessed using variation partitioning as well as distance-based redundancy analysis (db-RDA). Co-occurrence networks were formed using NetworkX software (version 2.3).

### Statistical analysis

We carried out the statistical analysis with the application of SPSS v20 software (IBM, Armonk, NY, United States). In addition, as all the tests were two-sided, the significance level was determined at 5%. Meanwhile, descriptive statistics was adopted for children’s sociodemographic features and clinical measurements. We used Student’s *t*-test and paired t-test to examine the significance of the difference in continuous variables along with alpha diversity between the two groups. In addition, the chi-square test was used to examine the differences in the discontinuous numerical variables between the two groups involved. To evaluate the correlation of the associated elements with the relative abundance of each species, Spearman’s correlation analysis was used.

## Results

### General characteristics of participants

16S rRNA gene sequencing of saliva samples was performed in a total of 55 infants, including the preterm (*n* = 31) and full-term control (*n* = 24) groups. We also compared all samples in the following four subgroups: preterm birth group before teeth eruption (PT0, 31 infants), preterm group after teeth eruption (PT1, 31 infants), control group before teeth eruption (CT0, 24 infants), and control group after teeth eruption (CT1, 24 infants).

The babies of the two groups presented no obvious differences regarding gender (*p =* 0.439) and enrollment month age (PT0 versus CT0, *p =* 0.940). Moreover, 11 out of 24 and 25 out of 31 babies were born by cesarean section (*p* < 0.001). The babies in the PT1 group were notably older (age in months) than the CT1 group (8.71 ± 1.37 versus 6.88 ± 1.30, *p* < 0.001), indicating a later teeth eruption age in preterm infants. Mean birth weight was significantly lower in the preterm group (1.67 ± 0.31) than in the control group (3.35 ± 0.39, *p* < 0.001). In addition, the mean weeks of gestation were significantly lower in the preterm group than in the control group (31.88 ± 2.51 versus 40.21 ± 0.52, *p* < 0.001). Table 1 shows the demographic characteristics of all participants. The average length of stay of preterm infants in an incubator and hospital was 31 and 20 days, respectively.

**Table 1:** Demographic characteristics of the participants.

		Preterm birth	Full term birth	*p*-values
		***N*** **(%)**	***N*** **(%)**	
Baseline	Age (months)			0.940
	Mean ± SD	1.06 ± 0.37	1.01 ± 0.03	
	Gender			0.439
	Man	19 (61.3)	13 (54.2)	
	Female	12 (38.7)	11 (45.8)	
	Birth weight (kg)			0.000
	Mean ± SD	1.67 ± 0.31	3.35 ± 0.39	
	Gestational weeks			0.000
	Mean ± SD	31.88 ± 2.51	40.21 ± 0.52	
	BMI (at delivery)			0.002
	Mean ± SD	21.83 ± 0.81	24.84 ± 0.95	
	Delivery mode			0.003
	Caesarean	25 (80.6)	11 (45.8)	
	Vaginal delivery	6 (19.4)	13 (54.2)	
Teeth eruption				
	Age (months)			0.000
	Mean ± SD	8.71 ± 1.37	6.88 ± 1.30	

*P*-values: Acquired using Chi-square test, Student’s t-test (two groups), or Fisher’s exact test; SD, standard deviation; BMI, body mass index; N, number of children examined.

In addition, there were no apparent differences in either group with respect to maternal demographic and socioeconomic characteristics, including age and education level, and oral hygiene and infant feeding habits at follow-up (including frequency of consumption of sweets; *p* > 0.05 for all). The results of the questionnaires are shown in Supplementary Table S1.

### Sequencing data

Altogether 4,278,926 high-quality reads were formed in 110 saliva samples. The average length was 455. After subdividing all samples to the same sequencing depth, sequencing and notation of OTUs (at the 3% divergence level) identified a total of 13 phyla, 31 classes, 58 orders, 107 families, 196 genera, 372 species, and 849 OTUs. Moreover, the rarefaction curve suggested the near-complete sampling of the saliva community (Supplementary Figure S1A). According to the core analysis curve, enough sequencing samples were obtained for this study (Supplementary Figure S1B). Using Venn diagrams in Supplementary Figure S1C, the number of shared and unique OTUs for the four subgroups is shown. We obtained and shared most OTUs between groups. Groups PT0 and PT1 shared 308 OTUs, and groups CT0 and CT1 shared 366 OTUs.

The distribution of relative abundances of bacteria from the phylum to species level is shown in Supplementary Figure S2. In addition, there were 129 genera, 198 species, and 363 OTUs in PT0, which increased to 143 genera, 254 species, and 420 OTUs in the follow-up of preterm infants.

### Saliva microbiome profiles differ in preterm infants

To evaluate the alpha diversity of the saliva bacterial microbiome, the richness index (Chao) and diversity index (Shannon) were used. Apart from that, the Chao index presented a higher OTU richness in the preterm infants at baseline, although no significant difference existed (Figure 1B, CT0 versus PT0 *p =* 0.491). After tooth eruption, the preterm group had significantly higher OTU richness than the full-term group (Figure 1C, CT1 versus PT1, *p* < 0.001). Based on a comparative assessment of the Shannon index, the preterm group initially had significantly higher microbial diversity than the control group (Figure 1D, CT0 versus PT0, *p* = 0.031), and the difference was not significant after tooth eruption (Figure 1E, CT1 versus PT1, *p* = 0.559).

The overall microbial composition and structure between preterm and control groups were compared with the use of PCoA and ANOSIM following the weighted normalized UniFrac distance (observed at the OTU level). Significant differences in salivary microbiota structure were found between the two groups at two stages of dentition (Figure 1F, CT0 versus PT0, *p* = 0.001, R = 0.320; Figure 1G, CT1 versus PT1, *p* = 0.001, R = 0.152).

### The longitudinal view of microbiota dynamics during the first teeth eruption

**Figure 2:**
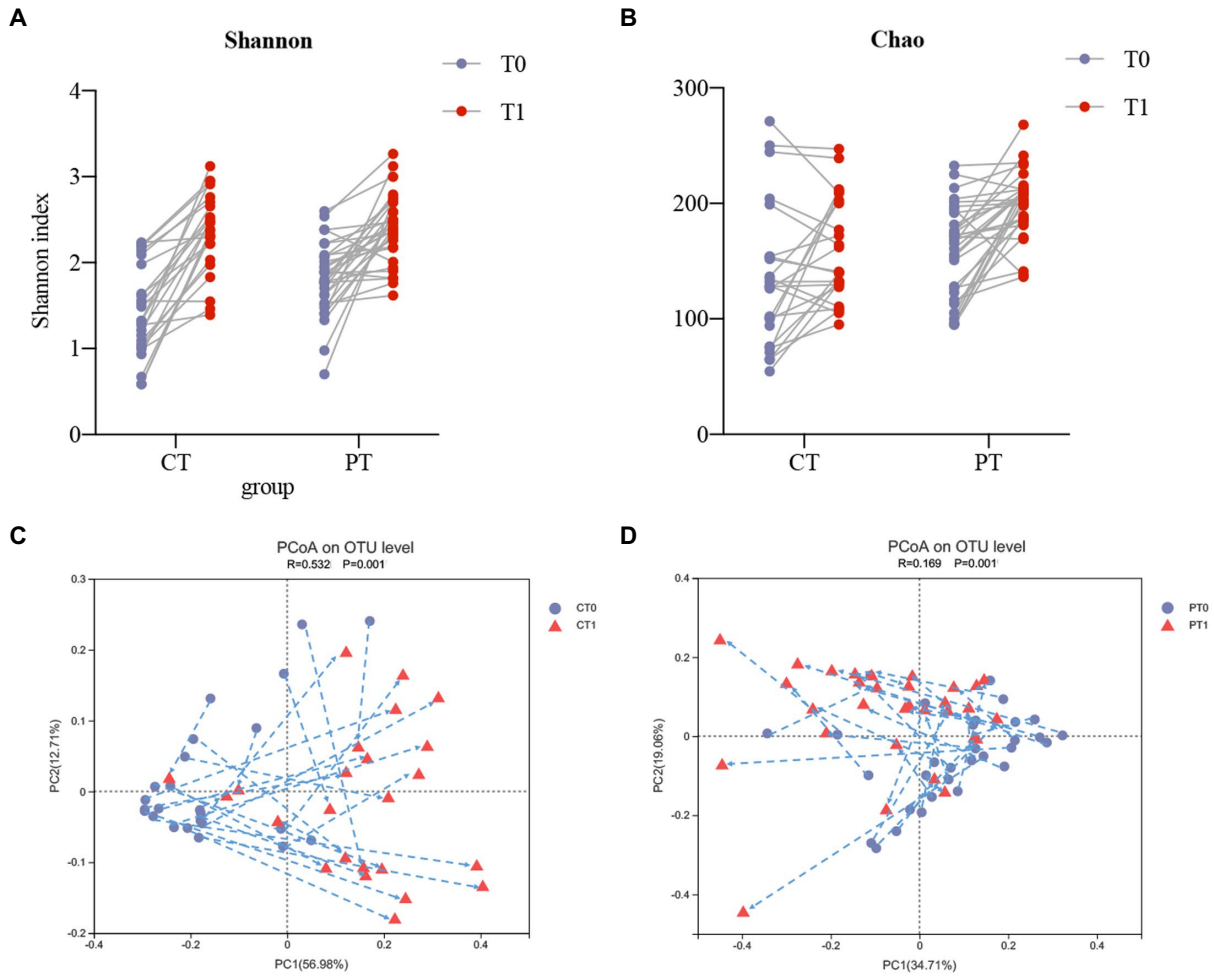
The longitudinal view of microbiota dynamics along with two lower primary central incisors teeth eruption. **(A,B)** Intraindividual shifts of bacterial richness and diversity shown by Shannon and Chao indices. **(C,D)** Intraindividual shifts of the microbiome community structure presented by PCoA, in accordance with the weighted normalized UniFrac distance, from baseline to the teeth eruption follow-up.

The comparative assessment of the Shannon and Chao indexes using paired t-tests revealed a generally statistically increased tendency in the alpha diversity in both full-term and preterm infants along with the first two lower primary central incisors eruption (Figure 2A, Shannon, CT0 versus CT1, *p* < 0.001, PT0 versus PT1, *p* < 0.001; Figure 2B, Chao, CT0 versus CT1, *p =* 0.031, PT0 versus PT1, *p* < 0.001). Of note, some individual samples had a lower Chao index and a higher Shannon index while growing up and after tooth eruption, suggesting a more balanced environment.

PCoA and ANOSIM confirmed intraindividual variations in the structure of the tongue-coating microbiota in all samples during tooth eruption and normal growth. According to the findings, the microbiome in the two groups revealed obvious shifting patterns (Figure 2C, CT0 versus CT1, *p =* 0.001, R = 0.532; Figure 2D, PT0 versus PT1, *p =* 0.001, R = 0.169). These preterm infants showed irregular variations in microbiome structures and shifted in various directions while growing up. Most of the samples in the control group could shift in the same direction. In addition, PC1 levels increased in 20 of 24 samples after tooth eruption, whereas PC2 levels decreased in 16 of 24 samples during follow-up.

### Variation in microbial composition

**Figure 3:**
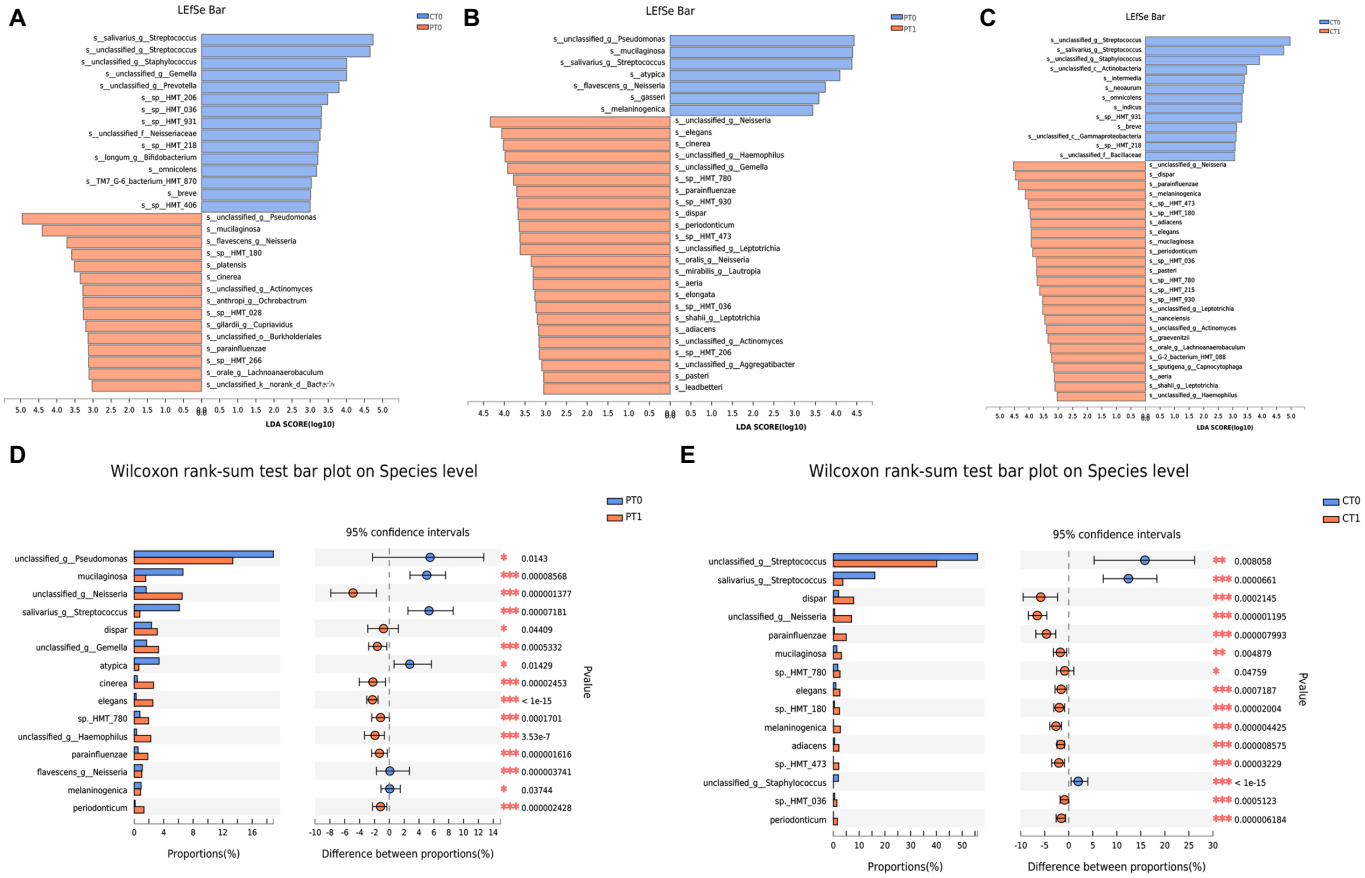
Variation in microbial composition. **(A–C)** Contrast of the relative bacterial abundances on the species level based on the LDA effect size (LEfSe) algorithm (LDA score [log10] >3). **(D,E)** Microbiome alterations at the species level along with two lower primary central incisors teeth eruption in preterm and full-term groups based on Wilcoxon rank-sum test. *p < 0.05, **p < 0.01, and ***p < 0.001.

To classify the microbiota associated with preterm birth and tooth eruption, LEfSe analysis and LDA scores at the species level were used to compare relative bacterial abundances between groups (Figure 3 and Supplementary Figure S3, LDA score [log10] >3). Compared with full-term infants, the early-stage microbiome in the preterm group was featured by 15 microbial biomarkers containing an unclassified *Pseudomonas* species, *Rothia mucilaginosa*, and *Neisseria flavescens* (Figure 3A). In addition, 15 bacterial species, including *Streptococcus salivarius, Staphylococcus* spp. and *Gemella* spp. were more abundant in the CT0 samples (Figure 3A). In the preterm infants that were full-length predentates, *S. salivarius* accounted for more than 3.80% of the total microbiota and decreased dramatically to 0.76% after tooth eruption. Similarly, an unclassified *Pseudomonas* species, *R. mucilaginosa*, *Veillonella atypica*, and *N. flavescens,* also declined in PT1 compared to the predentate stage (Figure 3B). In contrast, 24 bacterial species were abundantly enhanced during teeth eruption (Figure 3B). Similar to the longitudinal changes in the preterm group, *S. salivarius* decreased markedly from CT0 to CT1. However, *Prevotella melaninogenica,* which decreased from PT0 to PT1, increased from CT0 to CT1 (Figure 3C).

**Figure 4:**
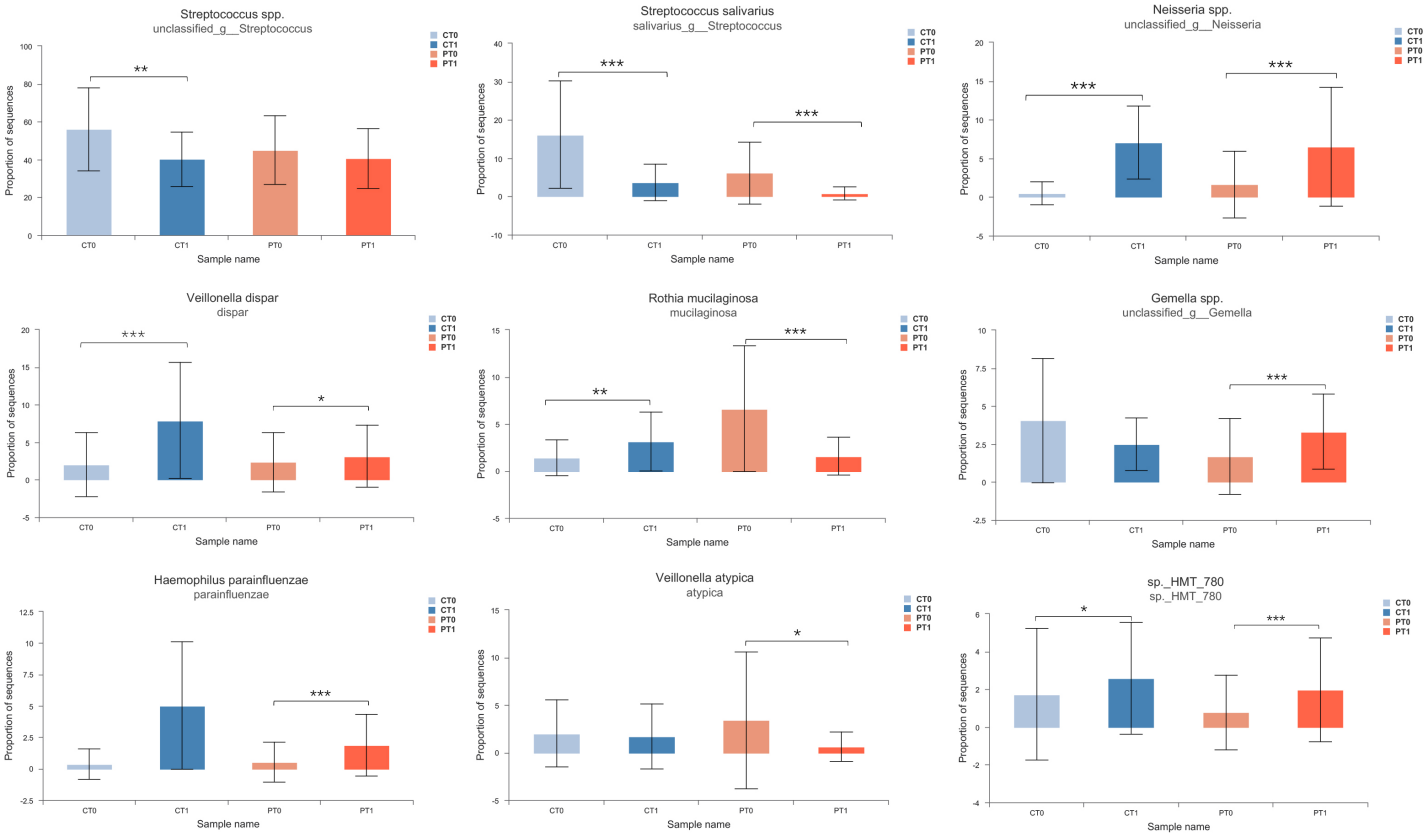
Variation of dominant bacterial species in infant saliva samples during teeth eruption. Relative abundance was compared using Wilcoxon rank-sum test. *p < 0.05, **p < 0.01, and ***p < 0.001.

In addition, the Wilcoxon rank-sum test was employed in the current work with the purpose of comparing the relative abundance of species in the saliva microbiota (Figures 3, 4, and Supplementary Figure S3). Notably, the relative abundance of *Gemella* spp. increased significantly (*p* < 0.001) and *V. atypica* decreased significantly (*p* < 0.05) in preterm infants at the rate of tooth eruption, whereas it remained relatively stable in healthy control subjects during tooth eruption. Longitudinal changes also showed that *R. mucilaginosa* decreased in preterm infants and increased in full-term infants. *Veillonella* spp. *oral taxon 780* (sp._HMT_780) and *Veillonella dispar* were both abundantly enhanced among preterm and full-term babies over time (Figures 3D,E, 4).

### The influence factors on saliva microbiome in preterm infants

We measured VIF to evaluate the collinearity among various environmental factors, such as body mass index (BMI) of mothers at delivery, gestational weeks, birth weight, breastfeeding habits, and incubator living days. Factors with a VIF >10 were excluded from the study. In addition, the VIF for maternal BMI, birth weight, duration of breastfeeding, length of hospital stay, and weeks of gestation for preterm infants was 1.32, 1.31, 1.00, 1.41, and 2.06, respectively. The VIF for days in the incubator was greater than 10 (VIF = 20.00) and was removed from the study.

**Figure 5:**
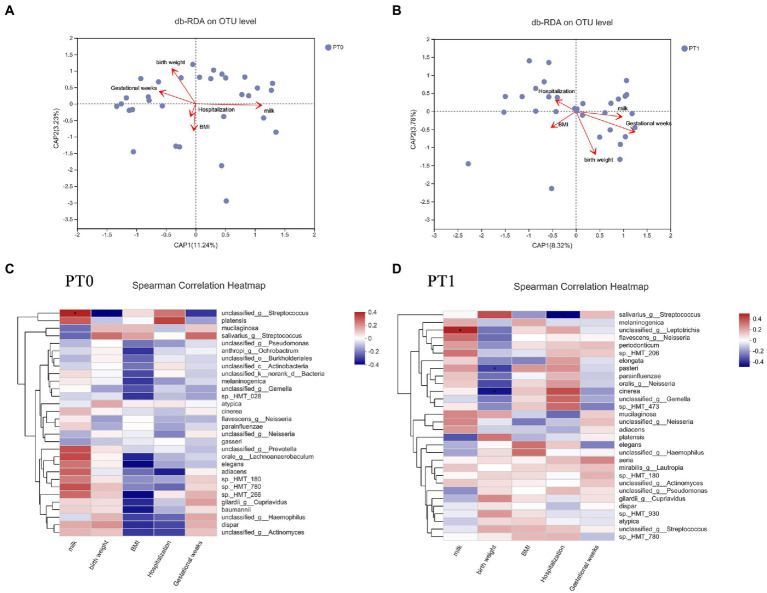
The influence factors on saliva microbiome in preterm infants. BMI, mothers’ body mass index; milk, breastfeeding durations; hospitalization, length of stay in the hospital at birth. **(A,B)** db-RDA plot at the OTU level in saliva samples. **(C,D)** Spearman’s correlation analyses for relative abundance (%) of the 30 most abundant species and associated environmental elements in saliva samples. *p < 0.05 and **p < 0.01.

The db-RDA on the basis of the weighted normalized UniFrac distance matrix was used for analyzing whether these environmental elements exerted any extra impact on the saliva microbiota communities and composition in preterm infants. At the predentation stage, birth weight and breastfeeding duration showed significant associations with salivary flora composition (Figure 5A, *r*^2^ = 0.34, *p* = 0.005; *r*^2^ = 0.39, *p* = 0.003, respectively). After teeth eruption, the db-RDA showed significant correlations between birth weight and gestational weeks at birth and the saliva bacterial community composition (Figure 5B, *r*^2^ = 0.34, *p =* 0.004; *r*^2^ = 0.30, *p =* 0.015, respectively).

In addition, Spearman’s correlation analyses were performed between the top 30 species (the relative abundance, %) in the salivary microbiota and these environmental factors in preterm infants (Figures 5C,D). At the predentate stage, a bacterial species of *Streptococcus* had a positive association with breastfeeding durations (*r* = 0.393, *p =* 0.028) as well as a negative relationship to birth weight (*r* = −0.363, *p =* 0.045). The relative abundances of *S. salivarius* (*r* = −0.473, *p =* 0.007) presented the reducing trend with the increasing length of stay in the hospital at birth in PT1 samples.

### Microbial correlation networks of saliva microbiota in preterm infants

A microbial correlation analysis was performed among the 30 most abundant species to investigate relationships in the salivary microbial community, finding apparent associations (*p* < 0.05) in the network.

**Figure 6:**
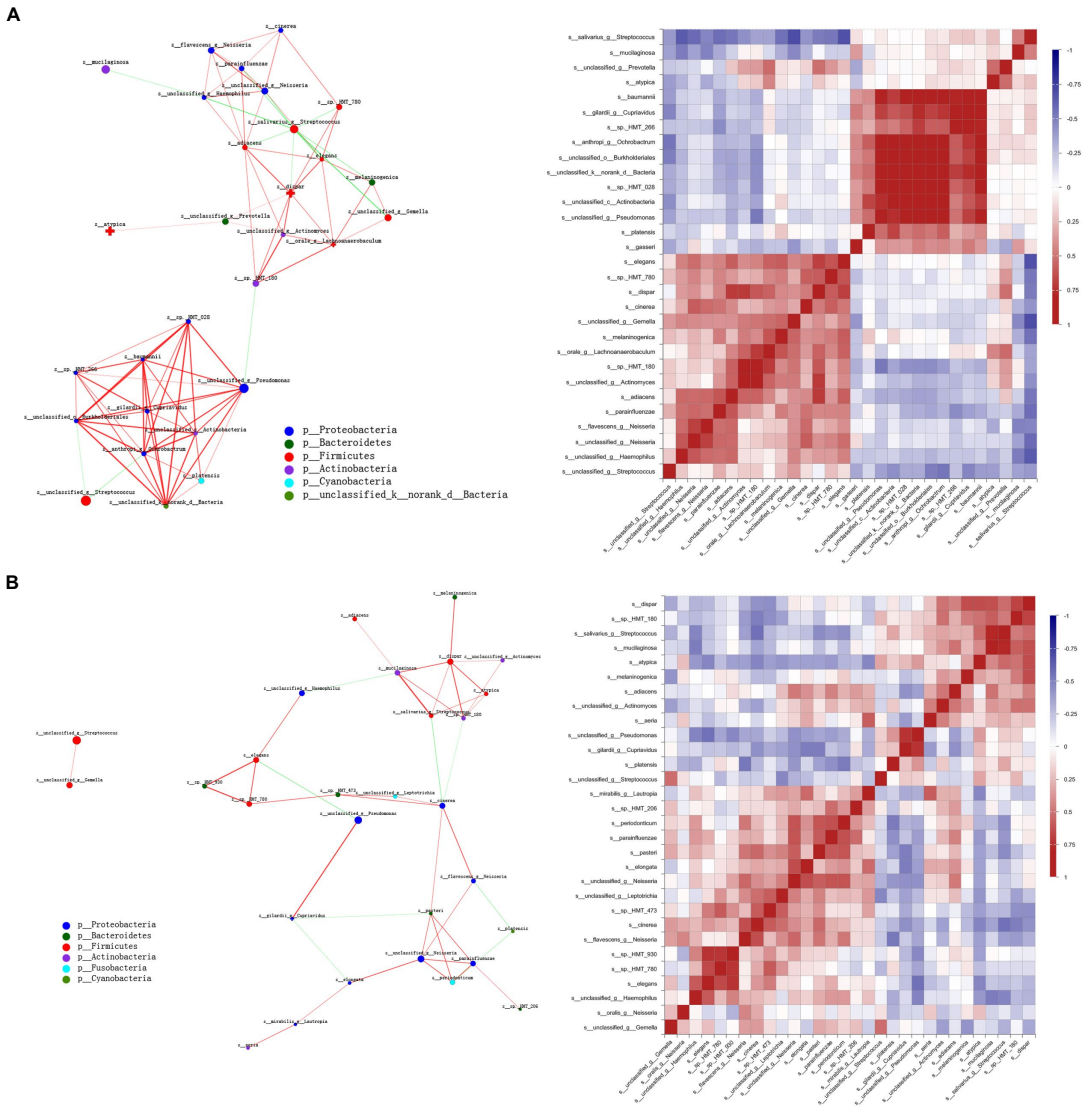
Microbial correlation networks of saliva microbiota in preterm infants. The network shows Spearman’s correlation coefficient calculation of 30 most abundant bacterial species together with significant associations (*p* < 0.05). Apart from that, the size of the nodes suggests the abundance of species, whereas the color of the connection reveals positive (red) and negative (green) correlations. **(A)** The PT0 group. **(B)** The PT1 group.

Remarkably, in this study, a cluster of interactions formed by *Pseudomonas* species with several members, including *Cupriavidus gilardii*, *Acinetobacter* spp. and *Acinetobacter baumannii*, was apparent in the predation phase. In addition, a dense hub of positive interactions was formed inside *Veillonella* and *Neisseria* species. Moreover, *S. salivarius* was negatively associated with *Haemophilus parainfluenzae*, *Prevotella melaninogenica*, *Gemella* spp., *Neisseria* spp., etc. (Figure 6A). At the tooth eruption stage, network analysis showed that there were still positive correlations between *Pseudomonas* (unclassified sp.) and *C. gilardii.* In addition, *S. salivarius* showed a strong positive correlation with *R. mucilaginosa, V. atypica,* and *V. dispar* (Figure 6B).

## Discussion

Microbiota colonization dynamics in infancy remains an interesting topic in microbial ecology and human health (Derrien et al., 2019). We know little about the growth of the infant oral microbial ecosystem (Del Chierico et al., 2015). The current work examined the effects of preterm birth on flora during a dental eruption. This study matched the month age of the two groups at baseline, which is a critical point and helps us understand the variations in the oral microbiome during tooth eruption.

The investigators reported an increase not only in bacterial diversity (Shannon) but also in richness (Chao) in the upbringing of both groups. The teeth eruption into the infant oral cavity starts at around 6 months of age in healthy infants and significantly later in preterm babies; thus, explaining a higher Chao diversity in PT1 than CT1 infants in this study. In the PT1 group, community development was more advanced. Mason et al. (2018) elucidated that oral microbiome in predentation infants is very similar to the maternal oral microbiome, and it drastically changes at the age of tooth eruption. The microbiome is also affected by the food diversity introduced along with milk (Mason et al., 2018). The CT0 and PT0 groups had distinctive microbial communities differing in the microbial structure at baseline and in shift patterns along with the teeth eruption period. PT0 showed irregular variations in microbiome structures. Tooth emergence can be related to ecological change. In addition, the tooth surface, gingival sulci, and interproximal spaces between teeth provide a wide variety of niches to develop highly complex microbiota and form a higher diversity of environments for microbial colonization and growth in the oral cavity (Jakubovics, 2015; Mark Welch et al., 2020).

In this study, the composition and displacement trend, as well as tooth eruption of oral nuclear flora differed in the full-term and preterm groups. In addition, an oral core microbiota composed mainly of *Streptococcus* spp. *salivarius*, *Pseudomonas* spp., *R. mucilaginosa, V. atypica*, *V. dispar, Gemella* spp. and *Neisseria* spp. (approximately more than 80.0% of the total oral microbiota) can be identified in the preterm group. While in the healthy birth group, *Streptococcus*, especially *S. salivarius*, *V. atypica*, *Gemella* spp. and *Staphylococcus* spp., dominated the infant oral microbiota. *Streptococcus* accounted for more than 70.0% of the total microbiota at the predental stage and then decreased to 44.9% at the CT1 stage. The obtained result is consistent with other studies (Dashper et al., 2019; Lif Holgerson et al., 2020). Cheema et al. (2022) have reported specific taxa containing *Streptococcus mitis*, *R. mucilaginosa*, *Veillonella parvula*, *S. salivarius*, *Gemella haemolysans,* and *Veillonella* HB016, which formed the infant core oral microbiota at the first 3 months of life. Dzidic et al. (2018) indicated *Streptococcus*, *Veillonella*, and *Lactobacillus* spp. to be “early colonizers” in saliva in the oral cavity. Mason et al. (2018) found that tooth eruption provides habitats for *Rothia*, *Fusobacterium,* and *Neisseria*, resulting in shifts in the core microbiome, and that the tooth environment could strongly influence the composition of the oral microbiome at different life stages relative to other factors. In addition, *Neisseria* spp. and *R. mucilaginosa* were more abundantly found in the healthy group because of teeth eruption. In preterm infants, *Neisseria* spp. increased notably, and *R. mucilaginosa* markedly decreased along with two lower primary central incisors teeth eruption. Gram-positive aerobic coccus *R. mucilaginosa* has been considered a normal oral microbiome, and it can play the role of the opportunistic pathogen leading to impairment, including septicemia and endocarditis under certain conditions (Maraki and Papadakis, 2015). The onset of tooth eruption may create new ecological niches in the oral cavity, leading to new surfaces of adhesion and thus favoring colonization by different flora. Whether the core microbiota shifts predominantly due to tooth eruption in preterm infants is still controversial.

The oral *Streptococcus* genus is the most comprehensively investigated genera of oral bacteria and controls the oral microbiome from birth until at least 7 years old (Sampaio-Maia and Monteiro-Silva, 2014; Dzidic et al., 2018). Because of the capability of adhering to and colonizing the mucosal surface lining, *Streptococcus* is especially abundant within the first months after birth and reduces with age (Sampaio-Maia and Monteiro-Silva, 2014; Dzidic et al., 2018). Xu et al. (2022) conducted a longitudinal study of the gradual eruption of deciduous teeth in infants and observed that *Streptococcus* accounted for more than 60% of the total microbiota and subsequently declined dramatically to 26.1% after teeth eruption, which is inconsistent with our results in both groups. *S. mitis* and *S. salivarius* species occupied the majority of oral streptococci (Dashper et al., 2019; Lif Holgerson et al., 2020). We observed that *Streptococcus* species were more abundant in the healthy controls than the preterm babies at early stages. Moreover, various findings demonstrated the reduction in *Streptococcus* abundance was accompanied by enhanced microbial diversity (Blum et al., 2022). In addition, this decreasing phenomenon of *Streptococcus* was observed in our study, which may be because of bacterial community development.

Microbial correlation analysis showed positive relationships between *Streptococcus* spp. and *Gemella* spp. and between *S. salivarius* and *R. mucilaginosa* at the PT1 stage. Members of *Streptococcus* can bind to other bacteria *via* surface adhesins, which can promote the development of certain bacterial communities, and occur together with *G. haemolysans*, and *R. mucilaginosa* (Kolenbrander et al., 2006; Nobbs et al., 2011; Sulyanto et al., 2019). In addition, the facultative anaerobic, Gram-positive diplococcus *G. haemolysans* primarily mucosal in mammals, particularly in the upper gastrointestinal tract with the oral cavity, and ferments free sugars into a mixture of acidic end products (Blum et al., 2022). This was associated with allergies in the oral microbiome of children (Dzidic et al., 2018), which were not found in our questionnaire analysis. Moreover, unlike the downward trend in the healthy group, *Gemella* spp. showed an upward trend along with two lower primary central incisors teeth eruption in preterm infants. As *Gemella* colonizes early, it helps in oral microbiome development relatively later in premature babies.

Correlation networks of saliva microbiota in preterm infants showed that *S. salivarius* had a strong positive correlation with *V. atypica and V. dispar* in the PT1 stage. Production of lactate from fermentation of free sugars by oral *Streptococcus* may be the essential nutrient for *Veillonella* spp. demonstrating synergistic interactions between the two species (Jakubovics, 2015). Other key bacteria, *Veillonella* spp. have also been considered early colonizers. The general uptrend of *Veillonella* spp. abundance was shown in several studies in healthy and preterm infants (Dzidic et al., 2018; Yin et al., 2021; Xu et al., 2022). In this study, the relative abundance of *V. dispar* also increased while growing up in both groups; however, *V. atypica* did not show this trend, which was stable in the healthy controls from CT0 to CT1 stage and decreased markedly in the preterm infants along with two lower primary central incisors teeth eruption. Metabolites produced by *Streptococcus*, including lactic acid, can pave the way for other microorganisms to colonize saliva, such as *Veillonella* (Gomez and Nelson, 2017). Early symbiosis in saliva may have ecological advantages over later symbiosis, promoting environmental changes through their metabolic products that are conducive to the growth of additional oral bacterial communities. An important consequence of the healthy development of the oral microbiome is the prevention of the most common childhood oral disease, dental caries. The early presence of oral pathogenic microorganisms in edentulous children may use saliva as a reservoir. However, the longitudinal impact of these initial colonizers on infant oral and systemic health is largely unknown and should be further investigated to provide useful information for active and passive immunization strategies against childhood oral diseases.

It is noteworthy that some species were detected only in the preterm group and were hardly present in the control groups, such as *Pseudomonas* spp. There was a high abundance of *Pseudomonas* spp. at baseline that decreased afterward, which may be because of hospitalization and antibiotic use of premature infants (Henares et al., 2021; Warniment et al., 2021). Common nosocomial genera, including *Staphylococcus*, *Acinetobacter*, and *Pseudomonas,* developed in infants subjected to antibiotics (Henares et al., 2021). A higher abundance of *Pseudomonas* spp. in the PT0 and PT1 groups indicated that preterm infants were more susceptible to oral or systemic infections.

In this study, birth weight and breastfeeding durations in preterm babies influenced the saliva microbiome at the predentate stage. Subsequently, birth weight and gestational weeks at birth presented a significant association with the bacterial community. Protective factors of breast milk, such as maternal sIgA, may stimulate colonization with commensal bacteria, which perform an essential function in fulfilling the critical tasks required to build a microbial community to establish a healthy microbiota profile in the oral cavity (Berger et al., 2020; Blum et al., 2022). Cheema et al. (2022) indicated that the ingestion of oligosaccharides from human milk was associated with infant oral and fecal taxa, while their direct clinical relevance was unknown. *Streptococcus* spp. was associated with breastfeeding duration in PT0. *Streptococcus* is one of the leading bacterial groups in human breast milk (Rodriguez, 2014; Boix-Amoros et al., 2016). In addition, the metabolic products (including lactic acid) that originated from *Streptococcus* species from the dietary oligosaccharides in breast milk can help in establishing other oral microorganisms, including *Veillonella* (Wernersson et al., 2006; Gomez and Nelson, 2017). The transmission of bacteria from breast milk, as well as the nutrients found at the vital time point in the growth of babies, might influence the colonization window of certain bacterial genera (Dzidic et al., 2018). According to our results, the duration of breastfeeding may induce variations in oral microbial succession patterns that persist over time. Consideration of neonatal treatment needs, including antibiotic use, is necessary depending on the physical condition, birth weight, and gestational weeks of preterm infants to exert a long-term influence on the oral bacterial composition.

Nevertheless, this work still has a few limitations. At first, the sample size was limited, particularly in the healthy control group. In this study, the enrollment and follow-up of the healthy babies were more challenging than the preterm babies. This may be because parents of babies without disease are less interested in participating in research projects, and parents of healthy babies often do not attend appointments; parents of premature babies, on the other hand, prefer follow-up appointments. Moreover, this study was conducted at only two childcare institutions, preventing us from obtaining a larger sample size. Second, this study followed up only two lower incisors’ eruption, which may limit the overall results of the study. Larger and longer longitudinal studies are required to explore the effect of oral microbial change because of the complicated oral microbial structures during teeth eruption. Third, because tooth eruption and the introduction of food in addition to milk can occur simultaneously [45], it is difficult to distinguish the effects of dietary habits and tooth eruption on the development of the oral microbial community. At last, the mode of birth delivery was not included in the bioinformatics analysis. Various researchers have compared colonization patterns of microbial species between infants delivered vaginally and those delivered by cesarean section. Hurley et al. (2019) reported that the effects of birth mode on infant oral microbiome development are short-lived. Infants delivered by cesarean section showed an obviously more diverse oral microbiota when compared with infants delivered vaginally at 1 week of age, whereas the existing difference did not persist after 4 weeks of birth. This study included infants at least 1 month of age, so there are no subgroups by mode of delivery. Considering that antibiotics are typically administered prophylactically during intrapartum care to reduce the risk of infectious complications during cesarean deliveries, which may confound microbiome results (Smaill and Grivell, 2014), a thorough investigation is needed to determine the effects of mode of delivery on the oral microbiome and whether they may persist over time.

## Conclusion

To conclude, this study provides certain knowledge on how the oral microbiota changes during teeth eruption in preterm newborns. Compared with full-term infants, the composition and shifting pattern, along with two lower primary central incisors teeth eruption, of core oral flora differed in preterm groups. Several microorganisms that populate the oral microbiome develop relatively late in preterm infants, such as *Gemella*. In addition to tooth eruption, the results of this study confirmed that breastfeeding duration and birth weight also influence the growth of the salivary microbiome in preterm infants.

## Data availability statement

The data presented in the study are deposited in the SRA repository, accession number PRJNA876796.

## Ethics statement

The studies involving human participants were reviewed and approved by the Ethics Committee at the Ninth People’s Hospital, School of Medicine, Shanghai Jiao Tong University. Written informed consent to participate in this study was provided by the participants’ legal guardian/next of kin.

## Author contributions

YZ and XC contributed to the study design, sample collection, clinical examination, data analysis, statistics, and interpretation, and drafting and critically revising the manuscript. G-ZC, Y-PW, and VF contributed to sample collection and drafting and critically revising the manuscript. X-PF contributed to the study design and drafting and critically revising the manuscript. All authors give final approval and agree to be accountable for all aspects of the work.

## Funding

This study was funded by the National Natural Science Foundation of China (No. 81800967), Shanghai Commission of Science and Technology (No. 22Y11903200), and Shanghai Municipal Health Commission (Nos. 2019SY027 and 2020YJZX0114).

## Conflict of interest

The authors declare that the research was conducted in the absence of any commercial or financial relationships that could be construed as a potential conflict of interest.

## Publisher’s note

All claims expressed in this article are solely those of the authors and do not necessarily represent those of their affiliated organizations, or those of the publisher, the editors and the reviewers. Any product that may be evaluated in this article, or claim that may be made by its manufacturer, is not guaranteed or endorsed by the publisher.

## Footnotes

^1^ http://rdp.cme.msu.edu/

^2^ http://drive5.com/uparse/
