# Supplementary material for: Microbiota of preterm infant develops over time along with the first teeth eruption
Source: Front Microbiol. 2022 Dec 22;13:1049021. doi: 10.3389/fmicb.2022.1049021 (PMC9813514; doi:10.3389/fmicb.2022.1049021)
Supplement: Supplementary file 1 [file Data_Sheet_1.docx]

**Supplementary materials**

**Microbiota of preterm infant develops over time along with teeth eruption**

**Yu Zhang, Yi-Pei Wu, Vivien Feng, Gui-Zhi Cao, Xi-Ping Feng, Xi Chen**

**Table S1**. Questionnaire information of the participants

|  |  | Preterm birth | Full term birth | *P-values* |
| --- | --- | --- | --- | --- |
|  |  | N (%) | N (%) |  |
| Baseline | Hospitalization durations |  |  | 0.000 |
|  | Mean ±SD | 30.97 ±17.91 | 3.21 ±1.41 |  |
|  | Incubator durations |  |  | 0.000 |
|  | Mean ±SD | 19.77 ±8.36 | 0.00 ±0.00 |  |
|  | Feeding |  |  | 0.004 |
|  | Breastfeeding | 9 (29.0) | 11(45.8) |  |
|  | Artificial feeding | 11 (35.5) | 0 (0.0) |  |
|  | Combination | 11 (35.5) | 13 (54.2) |  |
|  | Breastfeeding durations |  |  | 0.001 |
|  | Mean ±SD | 0.65 ±0.49 | 1.00 ±0.00 |  |
|  | Mothers’ education level |  |  | 0.865 |
|  | Middle School or below | 2 (6.5) | 1 (4.2) |  |
|  | High School | 1 (3.2) | 2 (8.3) |  |
|  | Vocational School | 3 (9.7) | 3 (12.5) |  |
|  | College | 20 (64.5) | 13 (54.2) |  |
|  | Graduate School | 5 (16.1) | 5 (20.8) |  |
|  | Fathers’ education level |  |  | 0.949 |
|  | Middle School or below | 1 (3.2) | 1 (4.2) |  |
|  | High School | 1 (3.2) | 1 (4.2) |  |
|  | Vocational School | 2 (6.5) | 3 (12.5) |  |
|  | College | 24 (77.4) | 17 (70.8) |  |
|  | Graduate School | 3 (9.7) | 2 (8.3) |  |
|  | Family’s monthly income per person, RMB* |  |  | 0.997 |
|  | ≤3000 | 1 (3.2) | 1 (4.2) |  |
|  | 3001–5000 | 9 (29.3) | 6 (25.0) |  |
|  | 5001–10 000 | 10 (32.3) | 8 (33.3) |  |
|  | 10 001–20 000 | 4 (12.9) | 4 (16.7) |  |
|  | >20 000 | 5 (16.1) | 3 (12.5) |  |
| Teeth eruption |  |  |  |  |
|  | Feeding |  |  | 0.562 |
|  | Breastfeeding | 3 (9.7) | 4 (16.6) |  |
|  | Artificial feeding | 9 (29.0) | 10 (41.7) |  |
|  | Combination | 19 (61.3) | 10 (41.7) |  |
|  | Breastfeeding durations |  |  | 0.000 |
|  | Mean ±SD | 2.35 ±0.80 | 4.21 ±1.61 |  |
|  | Toothbrushing frequency* |  |  | 0.389 |
|  | ≥2×/day | 6 (19.4) | 8 (33.3) |  |
|  | 1×/day | 4 (12.9) | 3 (12.5) |  |
|  | Not everyday | 17 (54.9) | 9 (37.5) |  |
|  | Night milk intake* |  |  | 0.874 |
|  | ≥1×/day | 25 (80.6) | 18 (75.0) |  |
|  | 1–6×/week | 3 (9.7) | 2 (8.3) |  |
|  | Seldom/never | 1 (3.2) | 2 (8.3) |  |
|  | Sweetened milk intake* |  |  | 0.486 |
|  | ≥1×/day | 0 (0.0) | 1 (4.2) |  |
|  | 1–6×/week | 2 (6.5) | 1 (4.2) |  |
|  | Seldom/never | 26 (83.9) | 20 (83.3) |  |
|  | Any sweet snack intake* |  |  | 0.980 |
|  | ≥1×/day | 20 (64.5) | 15 (62.5) |  |
|  | 1–6×/week | 8 (25.8) | 6 (25.0) |  |
|  | Seldom/never | 1 (3.2) | 1 (4.2) |  |

*P*-values: Acquired using Chi-square test, Student’s *t*-test (two groups), or Fisher’s exact test. *Some data are missing for the above variables. SD: standard deviation.

**
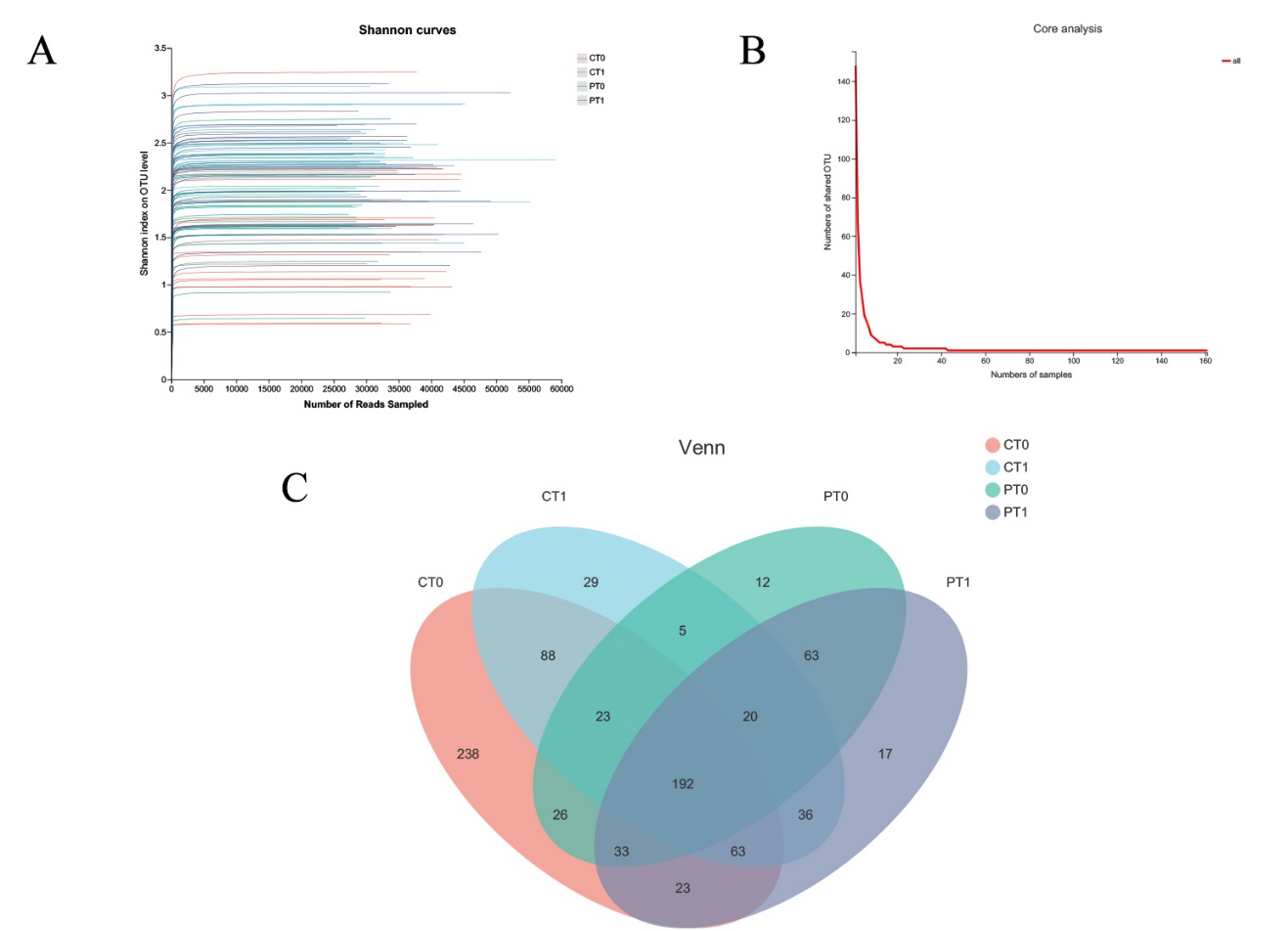
Figure S1.** **Sequencing data.** (A) Rarefaction curve. (B) Core analysis curve. (C) Venn diagram.

**
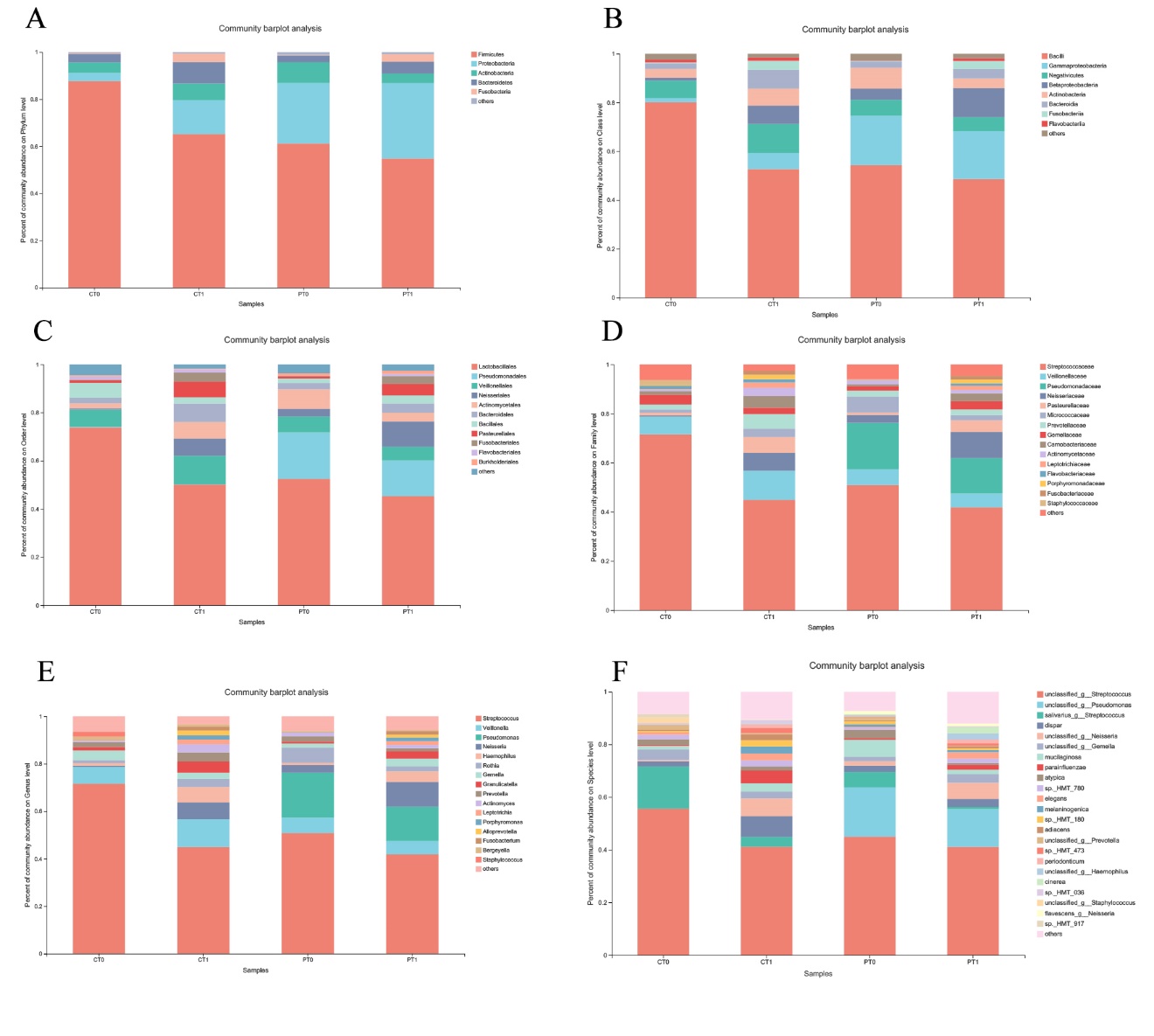
**

**Figure S2. Composition of bacterial communities in four subgroups from phylum to species levels.**

**
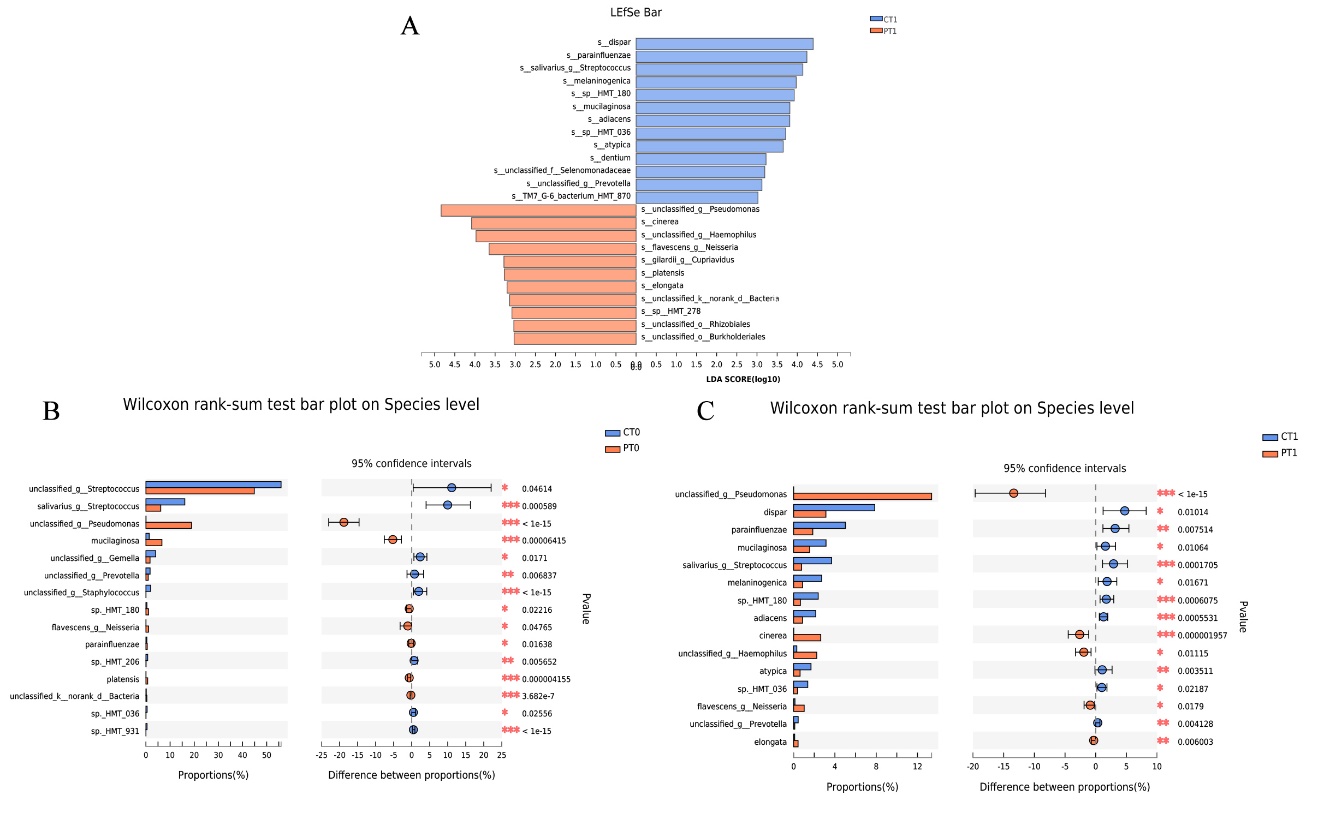
**

**Figure S3. Comparisons of the relative abundance of bacterial communities**. ^*^*P* < 0.05, ^**^*P* < 0.01, ^***^*P* < 0.001. (A) Contrast of the relative bacterial abundances on the species level based on the LDA effect size (LEfSe) algorithm (LDA score [log10] >3). (B, C) The Wilcoxon test was used for performing the comparisons of the relative bacteria abundance.
